# Supplementary material for: MicroRNA-mediated attenuation of branched-chain amino acid catabolism promotes ferroptosis in chronic kidney disease
Source: Nat Commun. 2023 Nov 28;14:7814. doi: 10.1038/s41467-023-43529-z (PMC10684653; doi:10.1038/s41467-023-43529-z)
Supplement: Supplementary file 1 — Supplementary Information [file 41467_2023_43529_MOESM1_ESM.pdf]

Supplementary Information

This PDF file includes Supplementary Figures 1 to 5 and Supplementary Table 1.

Supplementary Figure 1

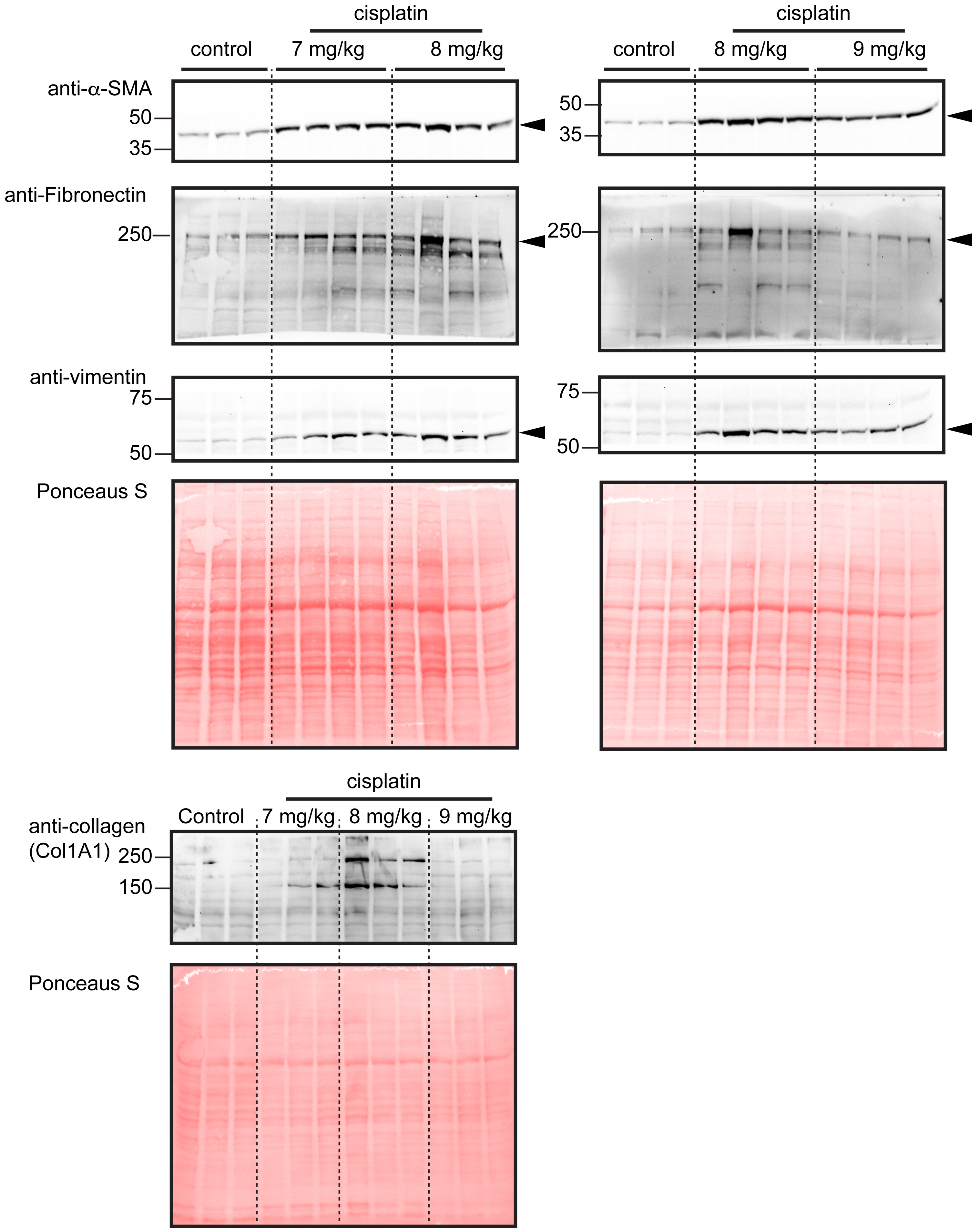

Supplementary Fig. 1 | Western blot analysis showing levels of the proteins (α-smooth muscle actin, fibronectin, vimentin, and collagen 1A1) associated with fibrosis in protein lysates from mice treated with cisplatin (7, 8, or 9 mg/kg). Ponceau S staining was used to determine equal protein loading per lane.

Supplementary Figure 2

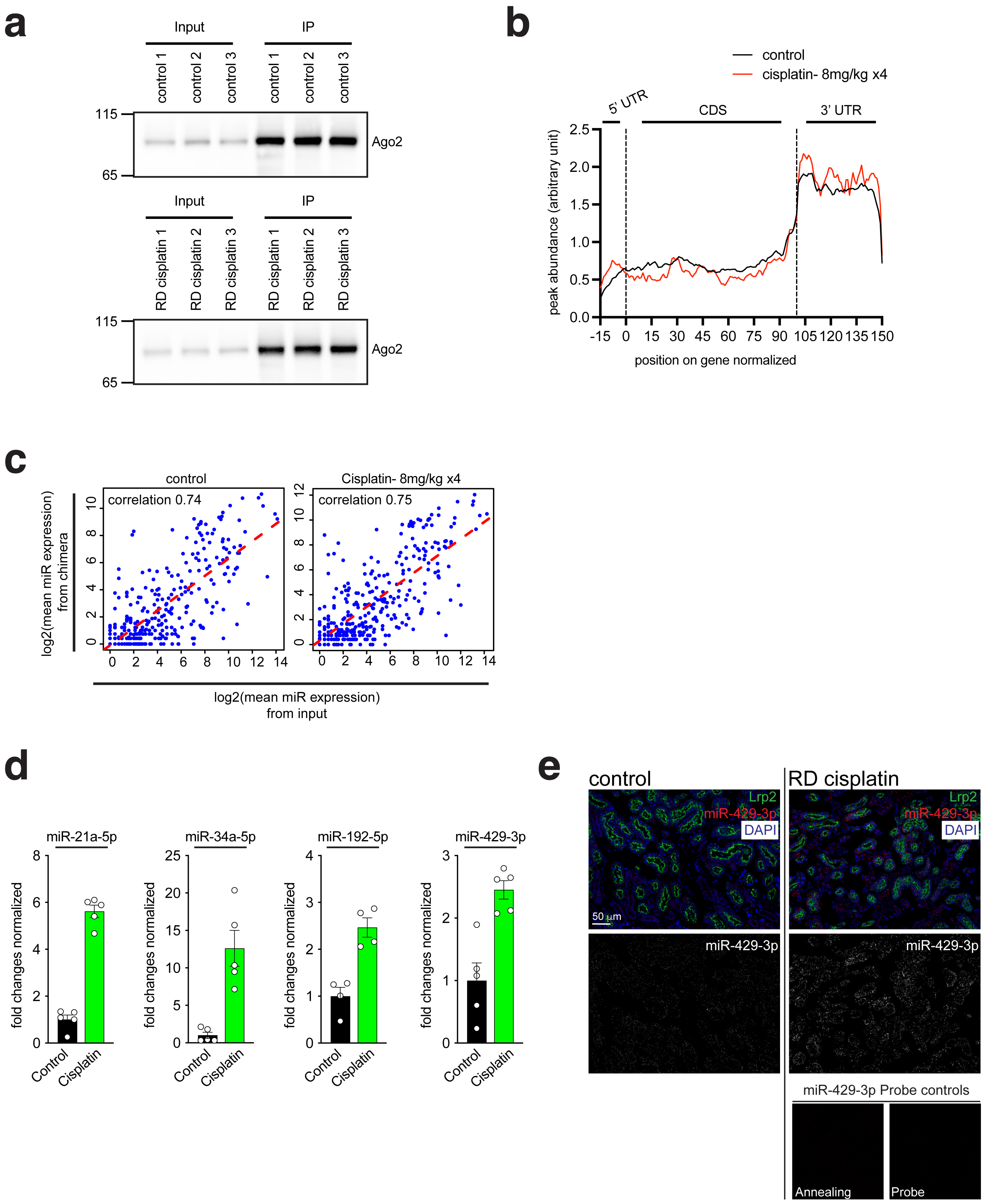

Supplementary Fig. 2 | chimeric-eCLIP-seq analysis in the cisplatin-injured mouse kidney. a. Immunoprecipitation of UV-crosslinked Ago2 in the presence or absence of cisplatin treatment. Western blot analysis shows successful Ago2 pulldown. **b.** The position of miRNA reads mapped along the metagene of mRNA from the chimeric-eCLIP-seq performed with UV crosslinking. 5'UTR, 5' untranslated region. CDS, coding sequence. 3'UTR, 3' untranslated region. **c.** Plots showing the correlation of miRNA reads obtained from chimeric-eCLIP-seq reads (*y* axis) and small RNA-seq (*x* axis). Each dot represents individual miRNAs. **d.** RT-qPCR analysis of four miRNAs from kidney samples, miR-21a-5p, miR-34a-5p, miR-192-5p, and miR-429-3p, to validate chimeric-eCLIP-seq results showing the cisplatin-induced increase in expression. Expression levels of the indicated miRNA were normalized using U6 snoRNA within each group, and then relative expression values of miRNA of the cisplatin-treated group normalized to those of the control group were plotted. Data are presented as Mean ± SEM. **e.** RNAScope analysis of miR-429-3p expression in cisplatin-induced kidneys, with Lrp2 as a proximal tubule marker and DAPI as a nucleus marker. Note that miR-429-3p was induced in the proximal tubules. No miR-429-3p signals were detected under the same imaging acquisition condition when a higher annealing condition or a control probe was used. Source data are provided as a Source Data file.

Supplementary Figure 3

a

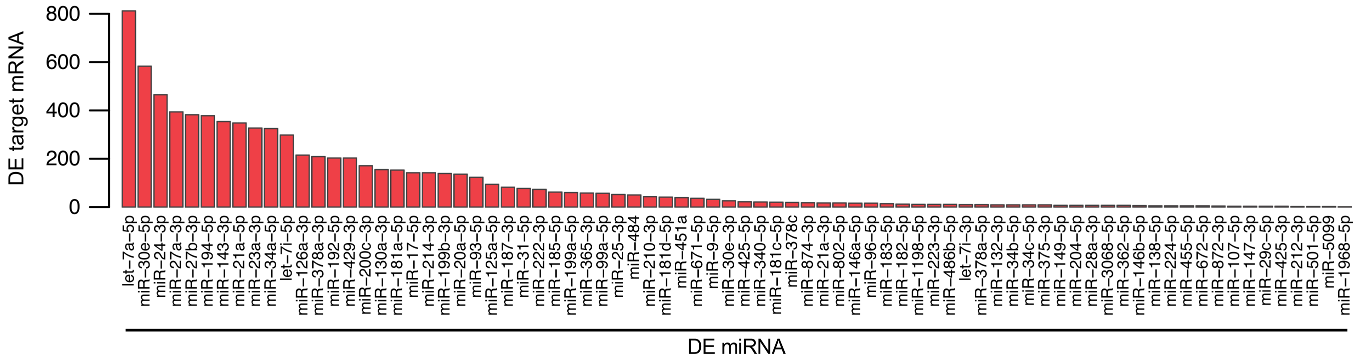

b

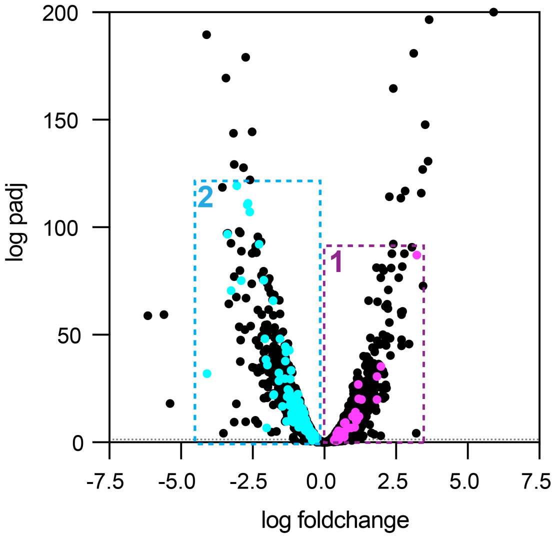

Group 1

metabolism	KEGG adjusted P value
Glutathione metabolism	p_{adj} = 9.33E-07
Fatty acid metabolism	p_{adj} = 0.0004
CytP450-drug metabolism	p_{adj} = 0.0007
Platinum drug resistance	p_{adj} = 0.001

Group 2

metabolism	KEGG adjusted P value
TCA cycle	p_{adj} = 9.50E-19
BCAA catabolism	p_{adj} = 2.40E-14
Oxidative phosphorylation	p_{adj} = 2.35E-11
Propanoate metabolism	p_{adj} = 1.40E-10
beta-alanine metabolism	p_{adj} = 7.78E-5

c

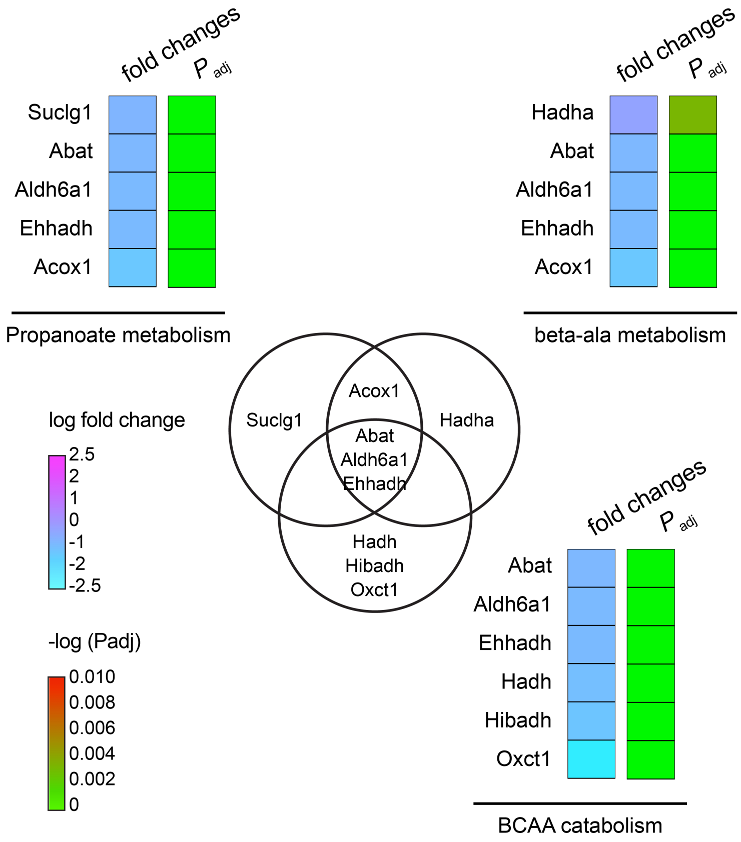

Supplementary Fig. 3 | Pathway analysis of target mRNAs of the miRNAs in the cisplatin-injured kidney. **a.** Display of miRNAs ranked by the size of their target miRNA sets. **b.** Volcano plot analysis of the fold changes of mRNA expression in response to cisplatin (*x* axis, (+) cisplatin / (-) cisplatin) combined with statistical significance (Padj) (*y* axis). Black dots represent individual mRNAs of total RNA-seq reads. The colored dots represent the individual target mRNAs identified by chimeric-eCLIP-seq. Group 1, GO terms with increased expression of target mRNA in response to cisplatin. Group 2, GO terms with decreased target mRNA expression in responses to cisplatin. Note the specific enrichment of GO terms by the direction of target mRNA expression change. *p* values are FDR-adjusted from the one-sided hypergeometric test, ShinyGO. **c.** Three metabolic pathway GO terms analyzed for shared mRNA elements. Note that GO terms for propanoate metabolism, β-alanine metabolism, and BCAA catabolism share a common set of target mRNAs (Venn diagram) of which expressions decrease in response to cisplatin. Source data are provided as a Source Data file.

Supplementary Figure 4

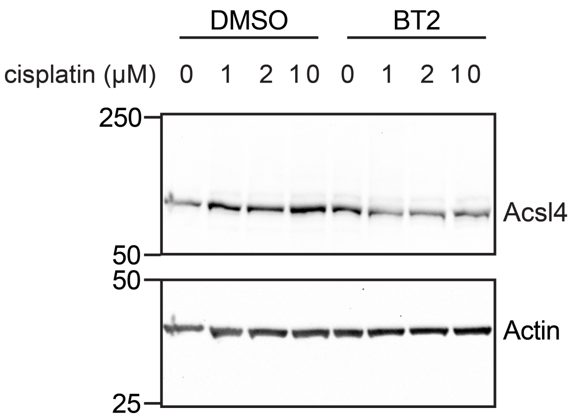

Supplementary Fig. 4 | Representative immunoblots of Acsl4 and actin protein expression in BUMPT cells, treated with indicated concentrations of BT2, with and without cisplatin exposure, across three independent experiments. Note that BT2-treated BUMPT cells expressed significantly reduced levels of Acsl4 protein in response to either 1 or 2 μM cisplatin but not considerably to a higher 10 μM cisplatin. β-actin used as a loading control. The source data for the quantification result shown in Figure 5c are provided as a Source Data file.

Supplementary Figure 5

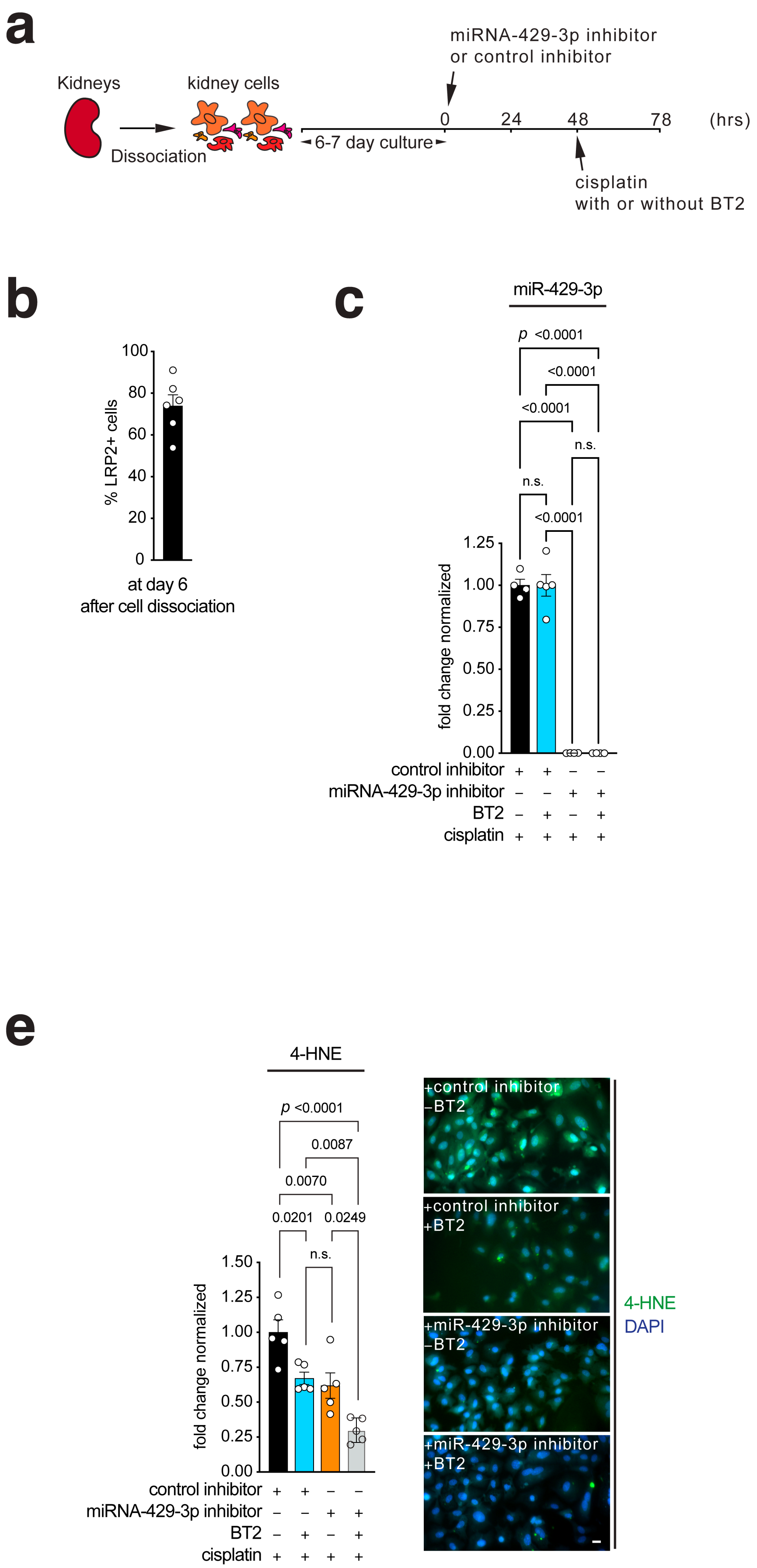

Supplementary Fig. 5 | The effect of miR-429-3p and BT2 in BCAA catabolism-associated ferroptosis. a. Schematic overview of the experimental procedure. **b.** LRP2 expression in dissociated kidney cells, determined by immunofluorescence. To automatically count the percentage of LRP2-positive cells in dissociated cells grown for 6 days using the EVOS M5000 imaging system, two parameters—LRP2 and DAPI intensity and area—were selected for object counting. Data are presented as Mean ± SEM, from six independent experiments. **c. and d.** RT-qPCR analysis of miR-429-3p (c) and Acsl4 (d) in the dissociated kidney cells treated with the indicated combinations of miRNA-429-3p inhibitor and BT2. Expression levels of miR-429-3p were normalized using U6 snoRNA, while expression levels of Acsl4 were normalized using Gapdh within each group. Their relative expression values normalized to those of the control group (cisplatin with control inhibitor) were plotted. Data are presented as Mean ± SEM, from four independent experiments. n.s., not significant ($p > 0.05$). P-values are from multiple comparisons of an ordinary one-way ANOVA test. **e.** Immunofluorescence analysis of 4-HNE expression in the dissociated kidney cells treated with the indicated combinations of miRNA-429-3p inhibitor and BT2. The intensities of 4-HNE signals from the fixed cells were normalized to area and nucleus number, and then the relative expression values, normalized to those of the control group (cisplatin with control inhibitor), were plotted. Data are presented as Mean ± SEM, with at least 6,000 cells per data point obtained from two independent experiments. n.s., not significant ($p > 0.05$). P-values are from multiple comparisons of an ordinary one-way ANOVA test. Scale bar, 20 µm. Source data are provided as a Source Data file.

Supplementary Table 1 | Materials used.

Primers

Gene	For	Rev	PMID (Ref)
Cxcl12	CATCCATCCATCCATCCA	TTCAGGGTCATGGAGACAGT	31249871
Cx3cl1	TCGGACTTTGTTGGTTCCTC	CAAAATGGCACAGACATTGG	31249871
Mif	AGTAAGCTGCTGTGTGGCCTGCT	CAGGACTCAAGCGAAGGTGGAAC	31249871
Bax	CCGGCGAATTGGAGATGAACT	CCAGCCCATGATGGTTCTGAT	34686727
Bcl-2	GCTACCGTCGTGACTTCGC	CCCCACCGAACTCAAAGAAGG	34686727
Gpx4	CCGGCTACAATGTCAGGTTT	ACGCAGCCGTTCTTATCAAT	33558472
Acsl4	ACTGGCGATATTGGAGAAT	CACATAGGACTGGTCACTT	35180475
Tfrc	CCCATGACGTTGAATTGAACCT	GTAGTCTCCACGAGCGGAATA	34843656
Timp2	CTCGCTGTCCCATGATCCC	GCCCATTGATGCTCTTCTCTGT	35218571
Igfbp7	AAGAGGCGGAAGGGTAAAGC	TGGGGTAGGTGATGCCGTT	35752325
Col1a1	TAAGGGTCCCCAATGGTGAGA	GGGTCCCTCGACTCCTACAT	28593951
Fn1	TGGTGGCCACTAAATACGAA	GGAGGGCTAACATTCTCCAG	24053613
Acta2	GTCCCAGACATCAGGGAGTAA	TCGGATACTTCAGCGTCAGGA	24591373
Vim	CCCTCACCTGTGAAGTGGAT	TCCAGCAGCTTCCTGTAGGT	24591373
Gapdh	ACCACAGTCCATGCCATCAC	TCCACCACCCTGTTGCTGTA	27724924
U6	GCTTCGGCAGCACATATACTA	CGAATTTGCGTGTCATCCTTG	30886169

LNA miRNA PCR assays

miRNA	Sequence	Catalog #
miR-21a-5p	5'UAGCUUAUCAGACUGAUGUUGA	Qiagen-339317
miR-429-3p	5'UAAUACUGUCUGGUAAUGCCGU	Qiagen-339317
miR-34a-5p	5'UGGCAGUGUCUUAGCUGGUUGU	Qiagen-339317
miR-192-5p	5'CUGACCUAUGAAUUGACAGCC	Qiagen-339317

Custom LNA miRNA inhibitors

miRNA	Sequence	Catalog #

Control		Qiagen-339137
miR-429-3p	5'T*T*A*C*C*A*G*A*C*A*G*T*A*T*T	Qiagen-339204
Control	5'T*A*A*C*A*C*G*T*C*T*A*T*A*C*G*C*C*C*A	Qiagen-339136
miR-429-3p	5'C*G*G*C*A*T*T*A*C*C*A*G*A*C*A*G*T*A*T*T	Qiagen-339130

RNAscope plus probes

miRNA	Catalog #
Control	ACD-smRNA RNA negative control probe for RNAscope Plus 4-plex
miR-429-3p	ACD-SR-mmu-miR-429-3p-S1
Lrp2	ACD-Mm-Lrp2-C2

Antibodies

Antibody (dilution used)	Vendor	Catalog #
Anti-acsl4 (1:1000 WB, 1:200 IF)	Thermo Fisher Scientific	PA5-27137
Anti-actin (1:2500 WB)	Sigma	A2228
Anti-smooth muscle actin (1:1000 WB)	Cell Signaling Technology	19245
Anti-fibronectin (1:1000 WB)	Abcam	ab2413
Anti-vimentin (1:800 WB)	Cell Signaling Technology	3932
Anti-collagen 1A1 (1:1000 WB)	Cell Signaling Technology	72026
LTL-FITC (1:500 IF)	Vector Laboratories	FL-1321-2
4-HNE (1:100 IF)	Japan Institute for the Control of Aging	MHN-100P
LRP2 (1:100 IF)	Proteintech	19700-1-AP

Chemicals

Chemicals	Vendor	Catalog #
BT2	Fisher Scientific	50-196-9375
Collagenase type II	Worthington Biochemical	CLS-2
Cisplatin	ZooPharm (now Wedgewood)	SKU-INJ020VC
