# Supplementary material for: MicroRNA-mediated attenuation of branched-chain amino acid catabolism promotes ferroptosis in chronic kidney disease
Source: Nat Commun. 2023 Nov 28;14:7814. doi: 10.1038/s41467-023-43529-z (PMC10684653; doi:10.1038/s41467-023-43529-z)
Supplement: Supplementary file 3 — Description of Additional Supplementary Files [file 41467_2023_43529_MOESM3_ESM.pdf]

Description of Additional Supplementary Files

Supplementary Data 1 | Total RNA-Seq (mRNAs).

Supplementary Data 2 | Small RNA-Seq (miRNAs).

Supplementary Data 3 | chimeric-eCLIP-Seq (miRNA:mRNA).

Supplementary Data 4 | Total RNA-Seq: LNA miR-429-3p inhibitor administration (mRNAs) and target mRNA list of miR-429-3p.
