# Supplementary material for: MicroRNA-mediated attenuation of branched-chain amino acid catabolism promotes ferroptosis in chronic kidney disease
Source: Nat Commun. 2023 Nov 28;14:7814. doi: 10.1038/s41467-023-43529-z (PMC10684653; doi:10.1038/s41467-023-43529-z)
Supplement: Supplementary file 8 — Reporting Summary [file 41467_2023_43529_MOESM8_ESM.pdf]

nature portfolio

Corresponding author(s): Sang-Ho Kwon

Last updated by author(s): Nov 6, 2023

Reporting Summary

Nature Portfolio wishes to improve the reproducibility of the work that we publish. This form provides structure for consistency and transparency in reporting. For further information on Nature Portfolio policies, see our Editorial Policies and the Editorial Policy Checklist.

Statistics

For all statistical analyses, confirm that the following items are present in the figure legend, table legend, main text, or Methods section.

n/a	Confirmed	
[ ]	[x]	The exact sample size (*n*) for each experimental group/condition, given as a discrete number and unit of measurement
[ ]	[x]	A statement on whether measurements were taken from distinct samples or whether the same sample was measured repeatedly
[ ]	[x]	The statistical test(s) used AND whether they are one- or two-sided<br>*Only common tests should be described solely by name; describe more complex techniques in the Methods section.*
[x]	[ ]	A description of all covariates tested
[x]	[ ]	A description of any assumptions or corrections, such as tests of normality and adjustment for multiple comparisons
[ ]	[x]	A full description of the statistical parameters including central tendency (e.g. means) or other basic estimates (e.g. regression coefficient) AND variation (e.g. standard deviation) or associated estimates of uncertainty (e.g. confidence intervals)
[ ]	[x]	For null hypothesis testing, the test statistic (e.g. *F*, *t*, *r*) with confidence intervals, effect sizes, degrees of freedom and *P* value noted<br>*Give P values as exact values whenever suitable.*
[x]	[ ]	For Bayesian analysis, information on the choice of priors and Markov chain Monte Carlo settings
[x]	[ ]	For hierarchical and complex designs, identification of the appropriate level for tests and full reporting of outcomes
[x]	[ ]	Estimates of effect sizes (e.g. Cohen's *d*, Pearson's *r*), indicating how they were calculated

Our web collection on statistics for biologists contains articles on many of the points above.

Software and code

Policy information about availability of computer code

Data collection	Western- iBright FL1000 (Thermo);<br>Imaging- EVOS M5000 (Thermo), Axio fluorescent microscope (Zeiss), STELLARIS confocal microscope (Leica);<br>qPCR- QS6 Pro (Thermo)
Data analysis	STELLARIS application suit (Leica); EVOS M5000 software, Rev 1.6 (Thermo); Fiji (Image J bundle, ver 2.9.0/1.53t;<br>QS Design and Analysis software 2 (Thermo);<br>fastQC ver 0.11.9; umi tools ver 11.2; cutadapt ver 4.1; STAR ver 1.7.10a; CLIPer; Bowtie ver 1.3.1; DESeq2 ver 4.2.3; R ver 4.2.2;<br>GraphPad Prism 10

For manuscripts utilizing custom algorithms or software that are central to the research but not yet described in published literature, software must be made available to editors and reviewers. We strongly encourage code deposition in a community repository (e.g. GitHub). See the Nature Portfolio guidelines for submitting code & software for further information.

Data

Policy information about availability of data

All manuscripts must include a data availability statement. This statement should provide the following information, where applicable:

- Accession codes, unique identifiers, or web links for publicly available datasets
- A description of any restrictions on data availability
- For clinical datasets or third party data, please ensure that the statement adheres to our policy

All data from the study are available in the main text or the supplementary materials. All sequencing data have been deposited in the NCBI Gene Expression Omnibus (GEO) under the series accession number GSE242809, which is publicly available. This series consist of CLIP-seq data (GSE242806), miRNA-seq data (GSE242807), and RNA-seq data (GSE242808).

Research involving human participants, their data, or biological material

Policy information about studies with human participants or human data. See also policy information about sex, gender (identity/presentation), and sexual orientation and race, ethnicity and racism.

Reporting on sex and gender	N/A
Reporting on race, ethnicity, or other socially relevant groupings	N/A
Population characteristics	N/A
Recruitment	N/A
Ethics oversight	N/A

Note that full information on the approval of the study protocol must also be provided in the manuscript.

Field-specific reporting

Please select the one below that is the best fit for your research. If you are not sure, read the appropriate sections before making your selection.

☒ Life sciences ☐ Behavioural & social sciences ☐ Ecological, evolutionary & environmental sciences

For a reference copy of the document with all sections, see nature.com/documents/nr-reporting-summary-flat.pdf

Life sciences study design

All studies must disclose on these points even when the disclosure is negative.

Sample size	The sample size for the animal experiments was not calculated. We included as many animals as possible in the experiments in order to achieve high statistical significance, taking into consideration factors such as variance and survival rates. We also took into account similar studies when determining the sample sizes.
Data exclusions	One control mouse was excluded for its death at the time of 2nd injection of cisplatin; One mouse treated with miRNA inhibitor was excluded for its death 1 day after the 4th injection of cisplatin.
Replication	All experiments, except for sequencing approaches and Fig S5c-e (two independent experiments for the BT2-treated group and three independent experiments for the other groups), were conducted with a minimum of three independent experiments. Additionally, for the RNA-seq approaches used in this study, we employed three biological replicates for each experimental group. All replication attempts indicated similar results.
Randomization	The mice used in this study were randomly divided into two groups: a control group and an inhibitor-treated group before starting cisplatin injection. Randomization was not required for the other experiments, and it was not performed for them.
Blinding	Different lab members conducted sample preparation and data analysis for RT-qPCR and Western analysis on the same samples. Sample identities were concealed using numerical IDs.

Reporting for specific materials, systems and methods

We require information from authors about some types of materials, experimental systems and methods used in many studies. Here, indicate whether each material, system or method listed is relevant to your study. If you are not sure if a list item applies to your research, read the appropriate section before selecting a response.

Materials & experimental systems

n/a	Involved in the study
☐	☒ Antibodies
☐	☒ Eukaryotic cell lines
☒	☐ Palaeontology and archaeology
☐	☒ Animals and other organisms
☒	☐ Clinical data
☒	☐ Dual use research of concern
☒	☐ Plants

Methods

n/a	Involved in the study
☒	☐ ChIP-seq
☒	☐ Flow cytometry
☒	☐ MRI-based neuroimaging

Antibodies

Antibodies used	anti-acsl4 (Rabbit, 1:1000 (WB), 1:200 (IF) Thermo PA5-27137)<br>anti-actin (Mouse, 1:2500, Sigma A2228)<br>anti-smooth muscle actin (Rabbit, 1:1000, Cell Signaling Technology, 19245)<br>anti-fibronectin (Rabbit, 1:1000, Abcam ab2413)<br>anti-vimentin (Rabbit, 1:800, Cell Signaling Technology, 3932)<br>anti-collagen 1A1 (Rabbit, 1:1000, Cell Signaling Technology, 72026)<br>LTL-FITC (1:500, VectorLabs, FL-1321-2)<br>anti-4HNE (Mouse, 1:100, Japan Institute for the Control of Aging, MHN-100P)<br>anti-LRP2 (Rabbit, 1:100, Proteintech, 19700-1-AP)
Validation	See manufacturer's information above.

Eukaryotic cell lines

Policy information about cell lines and Sex and Gender in Research

Cell line source(s)	BUMPT (Boston University C57BL/6 Mouse Proximal Tubule, clone 306), directly obtained from the Dong lab.
Authentication	No authentication of the BUMPT cell line was performed in my lab.
Mycoplasma contamination	BUMPT Cells used in this study were tested negative for mycoplasma contamination using LookOut mycoplasma PCR detection kit (MP0035, Sigma).
Commonly misidentified lines<br>(See ICLAC register)	No commonly misidentified cell lines were used.

Animals and other research organisms

Policy information about studies involving animals; ARRIVE guidelines recommended for reporting animal research, and Sex and Gender in Research

Laboratory animals	C57BL/6 (the Jackson Laboratory) 9-week old male mice. Mice were housed in temperature- (20-24°C) and humidity- (30%-70%) controlled, 12:12h light-cycled conventional animal quarters.
Wild animals	No wild animals were used in the study.
Reporting on sex	Sex as a biological variable was not taken into consideration in this study. Because female mice generally exhibit greater resilience to kidney damage compared to males, we initially focused on a male mouse model. As a result, we explicitly stated in the abstract, methods, and results sections that the research was exclusively conducted using male mice.
Field-collected samples	No field collected samples were used in the study.
Ethics oversight	The Medical College of Georgia IACUC

Note that full information on the approval of the study protocol must also be provided in the manuscript.
